# Editorial: Achieving Nutritional Security and Food Safety Through Genomics-Based Breeding of Crops

**DOI:** 10.3389/fnut.2021.638845

**Published:** 2021-02-04

**Authors:** Reyazul Rouf Mir, Ajay Kumar, Manish K. Pandey, Sachiko Narita Isobe

**Affiliations:** ^1^Division of Genetics and Plant Breeding, Faculty of Agriculture, Sher-e-Kashmir University of Agricultural Sciences and Technology, Srinagar, India; ^2^Depart of Plant Science, North Dakota State University, Fargo, ND, United States; ^3^Genomics & Trait Discovery Genetic Gains Program, International Crops Research Institute for the Semi-Arid Tropics, Patancheru, India; ^4^Department of Frontier Research and Development, Kazusa DNA Research Institute, Kisarazu, Japan

**Keywords:** genomics, nutrition, food safety, QTLs, genes, molecular breeding, varieties

The population of the world is continuously increasing and may exceed nine billion by 2050 (34% higher than today). To feed this increasing global population, the crop yields/food production must also increase by 70% by 2050 (1). Thus, the problem of hunger/malnutrition and demand for food security/healthier diets will be also on the rise (2). Although, we have achieved self-sufficiency in producing sufficient food (food quantity) in most countries in the world but the food produced is often deficient in essential nutrients (food quality) resulting in hidden hunger and malnutrition. Therefore, producing sufficient food is not a problem but producing quality food is a problem. The health of future generations will depend on our ability to produce and deliver food with enhanced nutrition for the growing population world-wide. The problem of micro-nutrient deficient food is a global problem now and this problem is seriously affecting people even in developed countries (1). Among nutrients that are most important and often deficient in diets include seed Zn, seed Fe and seed protein content (3, 4).

Conventional plant breeding methods have a long history of improving crop production, productivity, nutritional security, and food safety (5). Several nutrient dense crop varieties in different food crops have been released for cultivation world-wide. HarvestPlus in collaboration with different collaborating partners world-wide has developed several bio-fortified crops including Iron rich beans, iron rich pearl millet, vitamin A rich maize, zinc rich rice and zinc rich wheat and these crops have been released for cultivation in more than 30 countries (https://www.harvestplus.org/what-we-do/crops). In addition to improving nutrition security, the need of the hour is to also to address potential food safety issues related to allergenicity and toxicity of crop products prior to consumer consumption. The screening of crop germplasm for identification of genotypes with significantly reduced or null allergen content is cumbersome and therefore, conventional breeding methods and genomics assisted breeding (GAB) efforts toward hypoallergenic varieties have been undertaken in different crops including wheat, soybean, peanut, etc. (5).

The recent advances in genomic tools and techniques have revolutionized agriculture and changed the way food crop varieties are developed. A variety of genomics tools and techniques have been used in these crop improvement programs Such as high-throughput markers and marker-genotyping platforms, new genome mapping approaches, next-generation genome sequencing. Among genome mapping approaches, quantitative trait locus (QTL) mapping and genome-wide association mapping are two most important approaches being used for discovery of genes/QTLs for simple and complex quantitative traits (6). The advances in genome sequencing technologies have resulted in lowering the cost of sequencing by 100,000-fold since 2000 (7). The availability of next generation sequencing, high-throughput genotyping and high-throughput phenotyping technologies have completely changed the scenario and have increased the speed of germplasm characterization, allele mining, gene/QTL discovery and their use in marker-assisted breeding(MAB) programs (8). The application of genomics tools and techniques have resulted in the development of next-generation of crop varieties with enhanced yield potential and nutrition that is very critical for food and nutrition security and safety. Several modern breeding approaches including marker-assisted selection (MAS), marker-assisted backcrossing (MABC), marker-assisted recurrent selection (MARS) and genomic selection (GS) have been used for development of improved varieties in almost all important crop plants (6).

The current research topic entitled “**Achieving Nutritional Security and Food Safety Through Genomics-Based Breeding of Crops**” has a total of 17 articles including 13 research articles and four review articles. The articles have been written by authorities in their respective research fields. The articles provide great knowledge about the progress made in genomics for nutrition security in crops like rice, wheat, pearl millet, sorghum, chickpea, groundnut, mung bean, brassica, and radish. The articles deal with research areas that included evaluation of crop germplasm for important traits and development of genetic resources, mapping of genes through quantitative trait locus (QTL) interval mapping, QTL sequencing, association mapping including genome-wide association study (GWAS), QTL sequencing and genomics-assisted breeding (GAB) that included marker-assisted selection (MAS)/marker-assisted back crossing (MABC). Keeping in view the vast range of research areas covered in different manuscripts, we will first discuss the manuscripts that involved germplasm evaluation and development of genetic resources followed by manuscripts involving gene discovery through QTL mapping and association mapping and finally use of these QTLs/genes in development of improved cultivars through GAB approaches including MAS and MABC.

Peanut is one of the most important grain legume crops in the world and peanut allergy is considered serious health concern that affects more than 1% world's population. The allergy caused by peanut consumption is sometimes life-threatening that also affects life quality of allergic patients/their whole families. To alleviate this serious life threatening problem, development and consumption of hypoallergenic peanuts is considered the best solution. Therefore, efforts have been made and two important publications have been published in this research topic dealing with peanut allergy (Pandey, Varshney et al.; Pandey, Sudini et al.). The comprehensive manuscript by Pandey, Varshney et al. reported antibody based ELISA protocol for precisely quantifying and standardizing five major allergen proteins (Ara h 1, Ara h 2, Ara h 3, Ara h 6, and Ara h 8) in peanut seeds. The deployment of this protocol will allow large scale screening of peanut germplasm for selection of genotypes with minimum allergenicity to ensure food safety. In another study in peanut, Pandey, Varshney et al. used monoclonal antibody-based ELISA protocol to quantify five major allergens by phenotyping of highly diverse peanut germplasm panel. The analysis of trait data led to the identification of peanut lines with less allergenicity. This type of very useful peanut genetic resource will prove useful in future peanut improvement programs aimed at ensuring food safety while consuming peanuts. Similarly, in rice, one of the most important studies was conducted by Gurunathan et al. to develop rice mutant with enhanced resistant starch (RS) through the use of gamma rays as a mutagen. The development of resistant starch mutant will prove useful in human health and nutrition by controlling glucose metabolism. Further characterization of this mutant led to the identification of 31 sequence variants in six (GBSSI, SSI, SSIIa, SSIIIa, SBEIa, and SBEIIb) starch biosynthetic genes (Gurunathan et al.). In addition, three deleterious mutations/variants were also discovered in three starch biosynthetic genes including GBSSI, SSIIa, and SSIIIa with the potential to increase RS content in rice. In sorghum, the manuscript by Pandey, Madhu et al. showed the effectiveness of using antisense CYP79A1 strategy to produce transgenic sorghum plants with low cyanogenic potential, thus making sorghum safer for cattle feed. The desired sorghum plants having less cyanogenic potential were obtained by down-regulation of a key enzyme of dhurrin biosynthesis pathway “**cytochrome P450 CYP79A1**.”

Mapping of genes/QTLs for quantitative traits through QTL mapping and association mapping including genome-wide association studies (GWAS) is one of the most important subject areas of crop research. One of the important research articles published in this research topic on QTL mapping involving use of bi-parental mapping population segregating for seed micronutrients like seed Zn and seed Fe concentration is by Sab et al. The authors identified genes/QTLs for seed Zn and seed Fe concentration. For seed Fe concentration, eleven (11) QTLs were identified, while as for seed Zn concentration, eight (8) QTLs were identified. Among these identified QTLs, three QTLs were co-located in the “QTL-hotspot” region on linkage group-04 that also harbors several drought tolerance-related QTLs identified and reported in earlier studies. The knowledge gained in this study could be a helpful in the development of bio-fortified varieties in chickpea through genomics assisted breeding (GAB) approaches.

The other recently emerged mapping approach “**association mapping**” was used by Johnson et al. by involving use of high density SNP markers on a set of 243 durum wheat cultivars/advanced breeding lines developed over a period of 20 years. The results of this genome-wide association study (GWAS) revealed significant marker-trait associations for 24 nutritional/quality traits in durum wheat. The study identified several candidate markers that will help in durum wheat quality improvement through molecular breeding programs in future. Similarly, another study by Akhatar et al. reported use of genome-wide association study (GWAS) for discovery of genes/QTLs and dissect the genetics of oil, protein, and glucosinolates in Indian mustard (*Brassica juncea*). The study involved use of genotyping by sequencing (GBS) approach to identify genes for oil, protein, and glucosinolates. The authors further used meta-analysis of GWAS datasets to improve the power to recognize associations and identified common SNPs for oil and protein contents. In addition, 21 orthologs of the functional candidate genes related to the biosynthesis of oil, protein, and glucosinolates were also identified. The results of this study will prove useful in improving seed quality traits in *B. juncea* through marker-assisted selection (MAS) programs. In one of the important legume crops “**Mung bean**,” genome-wide association study (GWAS) were conducted by Wu et al. using a set of 6,486 SNPs markers identified through genotyping by sequencing (GBS) approach. The analysis of trait data and GBS data led to the identification of 43 significant marker-trait associations (MTAs) for seed calcium, iron, potassium, manganese, phosphorous, sulfur or zinc concentrations. The identified MTAs were distributed across 35 genomic regions explaining on average 22% of the variation for each seed nutrient. In another gene/QTL mapping study, Nayak et al. used single marker analysis (SMA) to identify significant marker-trait associations (MTAs). The study involved use of a set of 60 genotypes of peanut with different kernel color and size and data was recorded on nutritional traits including moisture, fat, ash, crude protein, crude fiber, carbohydrate content, and nutraceuticals including total polyphenol content and total antioxidant activity. The association analysis of trait data with the genotypic data led the identification of 75 major MTAs for most of the nutritional traits. The important markers found associated with nutritional traits and other important yield related traits will prove useful in genomics-assisted breeding (GAB) programs for developing nutrient-rich peanuts.

One of the important manuscript published in this research topic by Liu et al. reports combined use of most recently emerged gene mapping method “**QTL-Seq**” and **traditional linkage analysis** to identify candidate genes for purple skin of radish fleshy taproots. The study reported identification of one dominant gene “*Rsps*” followed by its fine mapping to a 238.51-kb genomic region containing 18 genes. These findings provide insight into the complex anthocyanin biosynthesis regulation in radish and information for molecular breeding to improve the anthocyanin content and appearance of radish taproots.

One of the ultimate and most important aims of germplasm evaluations/development of genetic resources and gene discovery programs is to identify best/suitable genes for their use in crop breeding programs to develop next-generation of crop varieties for cultivation by farmers. In this direction, some very good publications have been published in this research topic. For instance, in groundnut, Deshmukh et al. reported the use of marker-assisted selection (MAS) for introgression of high oleic content, resistance to late leaf spot and rust into popular groundnut cultivar “Kadiri 6.” The MAS was exercised for the alleles of target traits through the use of linked allele-specific SSRs and SNP markers. The study reported sixteen homozygous plants with high oleic, late leaf spot and rust resistance alleles. Similarly, Natesan et al. also used marker-assisted backcross breeding (MABB) approach in maize for increasing β-carotene concentration in parental genotypes of maize hybrid “**CO6**.” The MABC was exercised by involving the use of β-carotene gene “***crtRB1***” that resulted in the development of six improved maize lines with high recurrent parent genome recovery (90.24–92.42%), good agronomic performance and high β-carotene concentration (7.056–9.232 μg/g). The improved maize genotypes developed in this study will prove useful for ensuring food and nutritional security in the world. Govindaraj et al. reported fast-track intra-population genetic improvement for grain iron and zinc densities in pearl millet by involving three open-pollinated varieties. Progeny selection was found effective for Fe density and selection for Fe was found highly associated with the improvement of Zn density as well. Therefore, selection for one nutrient results in improvement of both the nutrients without yield penalty. This fast-track approach involves direct selection for additive genes and is therefore, important for bio-fortified breeding pipelines to address food-cum-nutritional security.

Four review articles were also published in the research topic. The review article by Gaikwad et al. provided comprehensive overview of literature on QTLs/Genes identified for seed Zn content, seed Fe content, seed proteins, vitamins, oil content, fatty acid content, and other nutritional traits in major food crops. Efforts have been also made to compile literature on recently emerged genomic approaches/genome editing technologies and their use in enhancing nutritional quality in major food crops. The review article by Rao et al. presented detailed information regarding bio-fortification for iron and zinc polished rice cultivars. The article discussed in detail the observations made from the characterization of rice germplasm including bi-parental mapping populations for seed zinc content/concentrations and development of national evaluation system for the release of bio-fortified rice varieties. Another important and comprehensive review by Mbanjo et al. on pigmented rice provided an update on the nutritional value and health benefits of pigmented rice. The review also describes different approaches including QTL mapping and association mapping being used for genetic dissection of pigmented rice. Efforts have been also made to provide insight into the mechanistic basis of grain pigmentation. The Philippines is one of the largest producers and consumer of rice in Asia and most of nutrition of people in Philippines is fulfilled by rice crop. The review article by Palanog et al. highlights the Zn deficiency in Philippines soil and its influence on the Zn nutritional status of the population. The article also highlights the efforts on rice bio-fortification that resulted in the development of two bio-fortified rice varieties approved for commercial release in the Philippines.

## Author Contributions

The manuscript was written by RM. AK, MP, and SI checked and approved the manuscript. All authors contributed to the article and approved the submitted version.

## Conflict of Interest

The authors declare that the research was conducted in the absence of any commercial or financial relationships that could be construed as a potential conflict of interest.
